# Does Heavy-Resistance Training Improve Mobility and Perception of Quality of Life in Older Women?

**DOI:** 10.3390/biology11050626

**Published:** 2022-04-20

**Authors:** Fernanda Borges-Silva, Miryam Martínez-Pascual, David Colomer-Poveda, Gonzalo Márquez, Salvador Romero-Arenas

**Affiliations:** 1 Faculty of Sport, Catholic University of Murcia (UCAM), 30107 Murcia, Spain; bsfernanda@ucam.edu; 2 Department of Cardiology, Hospital Universitario Los Arcos del Mar Menor, 30739 Murcia, Spain; miryammpascual@gmail.com; 3 Department of Physical Education, University of A Coruña, 15279 La Coruña, Spain; davcopo91@gmail.com (D.C.-P.); gonzalo.marquez@udc.es (G.M.)

**Keywords:** activities of daily living, mobility, resistance training, training intervention, walking speed

## Abstract

**Simple Summary:**

Heavy-resistance training can offer physicians a viable option for improving functional performance, helping their patients stay independent, active and healthy. The present results clearly demonstrate that circuit weight training is as effective as traditional resistance training for improving functional mobility but is more effective in stimulating positive perception of health-related quality-of-life adaptations in older women.

**Abstract:**

Regular physical exercise has shown great benefits in preventing age-related functional losses and in improving the perception of health-related quality of life (HRQoL) in older people. To optimize these benefits, it would be interesting to evaluate what type of exercise is better. Therefore, the purpose of this study was to assess the effects of heavy-resistance training on mobility and HRQoL in older women. Forty healthy, untrained older women (60–75 years) were randomly assigned to three groups: circuit resistance training (CRT, *n* = 15), traditional resistance training (TRT, *n* = 15) or the control group (CG, *n* = 10). During the 12-week training period, both experimental groups performed training with heavy loads, twice a week. Before and after the training period, the Timed Up and Go test, as a proxy of mobility, and the perception of HRQoL were evaluated. TRT and CRT resulted in a statistically significant improvement in the Timed Up and Go test (−5.4 and −10.3%, respectively; *p* < 0.05), but only the improvement after CRT was significantly greater than changes in the CG (*p* < 0.001). Only CRT elicited improvements in several dimensions of the perception of the HRQoL questionnaire, such as: physical functioning (13%, *p* < 0.001), general health (8.1%; *p* = 0.048), vitality (17.7%; *p* < 0.001), role emotional (6.7%; *p* = 0.044) and physical component summary (6.3%; *p* = 0.001). The change in the CRT group was greater than in the CG (*p* < 0.001) in the physical functioning score. The present findings show that CRT might be a time- (and hence cost-) effective alternative to trigger multiple positive functional and psychological adaptations in older women.

## 1. Introduction

The biological aging process is associated with a structural and functional deterioration of most physiological and psychological systems. These age-related changes affect a wide range of tissues, organ systems and functions that have a negative impact on activities of daily living in older adults [1]. Decreases in muscle strength and aerobic power are examples of functional declines with age, which can seriously limit a person’s physical performance and independence [2], the latter correlating with all causes of mortality [3,4,5,6,7]. Maintaining good health and physical fitness throughout the aging process is therefore a major challenge for healthcare systems and for aging people themselves [8].

The goal of “good health” can be qualified in different ways. The World Health Organization defined health as “a state of complete physical, mental and social well-being, not merely the absence of disease or infirmity”. This statement underscores the importance of overall quality of life for human well-being. Quality of life is an aspect of health experienced by subjects and could also be expressed as subjective health or health-related quality of life (HRQoL). Another factor, the ability to move around within one’s environment independently or with the help of an assistive device (i.e., mobility), is also associated with health and quality of life [9]. Natural aging and physical inactivity are conducive to the development of mobility impairments and, as a result, many older adults have difficulty walking and performing other activities of daily living [10].

Regular physical training prevents age-related losses in physical and functional performance and improves the perceived quality of life [8]. The American College of Sports Medicine’s current recommendations for the type and amount of physical activity healthy adults need to improve or maintain health include both cardio and resistance training [1]. One of the most common concurrent training methods is resistance-based circuit training, which simultaneously promotes aerobic conditioning, muscular endurance and strength adaptations [11]. This method involves single or multiple sets of different resistance training exercises performed sequentially with a very short rest period between exercises (e.g., 30 s). The exercises are performed at low, moderate or high loads, with a high number of repetitions (e.g., 12–15) or lower (e.g., <12) or with a specified period of time (e.g., 30 s) [12]. Thus, circuit resistance training is presented as a time-efficient modality that increases maximal oxygen consumption, maximal lung ventilation, functional capacity and strength while improving body composition [13].

Despite the importance of finding time-effective exercise strategies, such as circuit training, that improve different health-related factors, no studies have been found that assess the effect of a circuit resistance training program on mobility and the perception of HRQoL, two relevant factors of an individual’s health status. An overloaded resistance training program alone does not improve the perception of HRQoL [14]. Sillanpää et al. [14] observed that only resistance training improved the perception of HRQoL when combined with aerobic exercise. These authors observed that there were correlations between cardiorespiratory fitness and some of the dimensions of HRQoL Therefore, we hypothesized that a circuit training program, which has been shown to increase cardiorespiratory fitness, will also lead to improvements in the perception of HRQoL in older people. Therefore, the main aim of this study was to determine the effects of a supervised 12-week heavy-resistance training program on mobility and HRQoL in older women.

## 2. Materials and Methods

### 2.1. Participants

The study involved healthy, untrained women aged 60–75 living in southeastern Spain. The selection of subjects was carried out through a convenience sampling. Subjects without contraindications to physical activity were invited to a clinical examination. The initial anamnesis excluded the subjects who might have contraindications to resistance training with heavy loads (e.g., muscle or joint injuries, people with hypertension, among others) or pathologies affecting the results, such as depression or Alzheimer’s. The selected subjects were considered healthy, but none had any experience of systematic resistance or endurance training. The researchers organized different meetings in the community centers of the area to explain the characteristics of the project. All women interested in being part of the study voluntarily responded to the researchers’ call.

Participants provided written informed consent for the experimental procedures, which were approved by the university’s Institutional Review Board, and were also informed about possible injuries and benefits derived from the study. They were told that they could withdraw from the study at any time without giving a reason and without causing any harm to themselves. The study was conducted in accordance with the latest Declaration of Helsinki.

### 2.2. Design

This study was carried out between September and November 2021. Prior to data collection, participants attended a familiarization session for each test. During the familiarization session, subjects read the SF-36 questionnaire and any questions they had were answered. In addition, subjects walked around the room and sat down and got up from the chair they would later use for the test. One week after familiarization, the dependent variables were tested as described below. Participants were then randomly assigned to either circuit resistance training (CRT, *n* = 15), traditional resistance training (TRT, *n* = 15) or the control group (CG, *n* = 10). Participants were tested at weeks 0 and 13 by the same investigator using the same protocol and at the same time of day. The assessor was blinded; he did not know to which group each participant belonged. During the 12-week training period, both training groups (TRT and CRT) performed heavy-load resistance training (maximum of six repetitions (6RM; the load that induced failure after six repetitions) using Technogym Fitness equipment (Technogym S.A., Cesena, Italy), in undulating periodized programs twice a week with two rest days between each training session (i.e., Monday and Thursday or Tuesday and Friday). Resistance training sessions included lying leg curls, pec deck fly, seated calf raises, seated row, leg extension and preacher curl. All participants were asked to maintain their normal daily routines and eating habits, not take any dietary supplements that might impair cognitive or physical function, and not begin any new exercise programs for the duration of the study.

### 2.3. Assessments

#### 2.3.1. Health-Related Quality of Life

Health-related quality of life was assessed using the self-assessment questionnaire SF-36-Item Health Survey. SF-36 estimates HRQoL in eight separate dimensions related to physical, emotional and social well-being. The eight dimensions are physical functioning, role physical, bodily pain, general health, vitality, social functioning, role emotional and mental health. Subscale scores and summary scores (physical and mental components) were calculated using published algorithms [15]. Scores for each dimension ranged from 0 to 100, with a higher score reflecting better quality of life. The Spanish version of SF-36 is a valid and reliable method to measure HRQoL among the Spanish adult population [15].

#### 2.3.2. Timed Up and Go

Timed Up and Go (TUG) test is a timed version of the Get Up and Go test that attempts to address the subjectivity of the ordinal scale by introducing an objective measure, the total time to complete the task [16]. In the TUG, an examiner uses a stopwatch to record how many seconds the participants need to complete the task. Several studies have found that this continuous measurement is representative of a person’s walking ability, balance and risk of falling [17].

For the TUG test, the subject stood up from an armless chair, walked three meters, pivoted around a placed cone, walked back and sat down again. The time to perform the TUG test from rising from a sitting position to chair contact was recorded using a standard stopwatch. The best time of two trials was used for data analysis [18].

### 2.4. Intervention (TRT and CRT) and Control Groups

The intervention groups underwent a resistance training program that focused on both major and minor muscle groups, based on recommendations from the ASCM [1]. Prior to beginning the intervention, two sessions were used to familiarize the subjects with the exercises and to determine initial strength levels. Subjects were tested for their 6RM using previously described procedures [19]. Data obtained from the initial testing sessions were used to determine an appropriate starting resistance for week one of the study.

All training sessions started with a general warm-up (i.e., 10 min of walking at their usual pace and 5 min of joint mobility) and ended with a cool down with five minutes of stretching. After the general warm-up, participants performed a specific warm-up consisting of two sets of three exercises (lying leg curls, pec deck fly, seated calf raises) with the following sequence: 12 reps at 50% of 6RM (i.e., the load that led to failure after six reps), one minute of rest, 10 reps at 75% of 6RM, two minutes of rest and then the first main training set. The 6RM load was adjusted for the subsequent set by approximately 2% if a subject performed ±1 rep, or by approximately 5% if a subject performed ±2 reps. In each session, participants lifted weights that allowed only six repetitions (6RM, ~85–90% of 1RM). After completing the first three exercises, participants completed the other three exercises (seated row, leg extension and preacher curl) using the same specific warm-up sequence (~five min). Participants were supervised by an experienced lifter to control rest periods and ensure that volitional fatigue was safely achieved.

The TRT group performed the exercises sequentially with a three-minute break between sets. Total training time in the TRT group ranged from 45 min (one set) to 87 min (three sets). The CRT group differed from the TRT only in the rest interval between exercises and the sequence of exercises. While the TRT group performed the exercises sequentially with a three-minute break between each set, the CRT group completed the workout in two short circuits five minutes apart. The first circuit consisted of lying leg curls, pec deck fly and seated calf raises, while the second circuit consisted of seated rows, leg extensions and preacher curls. Approximately 35 s separated each exercise, allowing enough time to move safely between exercises. Warm-up and exercise intensity and volume were the same as in the TRT group. Total training time in the CRT group ranged from 35 min (one set) to 47 min (three sets).

Participants in the CG were asked to continue with their daily routine. The CG met at the training facility every three weeks for social gatherings with the researchers.

### 2.5. Statistical Analysis

Statistical analysis of data was performed with SPSS 26.0 for Windows (IBM Corp. Armonk, NY, USA). The physical characteristics of the participants are reported as means ± standard deviation. The normal distribution and homogeneity parameters were checked using Shapiro–Wilk and Levene tests, respectively. A two-way repeated measures analysis of variance (RM-ANOVA) with TIME (pre- to post-test) and GROUP (TRT vs. CRT vs. CG) as factors was performed to analyze the training-related effects. If there were statistically significant differences (*p* < 0.05) for the time factor, pairwise comparisons were performed to assess pre- to post-test differences for each individual group. Then, one-way ANOVAs with Tukey’s pairwise post hoc comparisons were performed on the pre- to post-trial change scores (normalized values relative to the pre-test). Significance was accepted when *p* < 0.05. Power (1-*β*) was determined for all variables, and effect sizes were reported as partial eta-squared ($\eta_{p}^{2}$) and Cohen’s d. The following criteria for effect sizes were used: <0.35 = trivial, 0.35–0.8 = small, 0.8–1.5 = moderate and >1.5 = large [20].

## 3. Results

Table 1 shows the descriptive characteristics of the participants in each group. No significant differences in training compliance between the TRT and CRT groups were found (96.6 ± 2.1 vs. 95.8 ± 1.9%, respectively). Supplementary Figure S1 reports on the evolution of loads for each exercise over the course of training. Although we did not take measures of pre- to post-maximal dynamic voluntary force (i.e., one repetition maximum), Supplemental Figure S1 clearly shows how participants in the TRT and CRT groups increased their muscle strength week by week, being able to lift more weight for the same number of repetitions on each set (the weight was adjusted to the maximum weight they could lift for six repetitions).

### 3.1. Health-Related Quality of Life

Table 2 shows the baseline and post-training values of the perception of HRQoL for the groups under study. Two-way RM-ANOVA revealed a significant effect of time for the TPT group in physical functioning (F = 6.697; *p* = 0.014; $\eta_{p}^{2}$ = 0.153; 1 − *β* = 0.712), but there were no changes in role physical (F = 2.908; *p* = 0.097; $\eta_{p}^{2}$ = 0.073; 1 − *β* = 0.383), bodily pain (F = 0.249; *p* = 0.621; $\eta_{p}^{2}\;$= 0.007; 1 − *β* = 0.077), general health (F = 3.095; *p* = 0.087; $\eta_{p}^{2}$ = 0.077; 1 − *β* = 0.403), vitality (F = 2.621; *p* = 0.114; $\eta_{p}^{2}$ = 0.066; 1 − *β* = 0.351), social functioning (F = 3.720; *p* = 0.061; $\eta_{p}^{2}$ = 0.091; 1 − *β* = 0.468), role emotional (F = 0.000; *p* = 1.000; $\eta_{p}^{2}$ = 0.000; 1 − *β* = 0.050), mental health (F = 1.467; *p* = 0.234; $\eta_{p}^{2}$ = 0.038; 1 − *β* = 0.219), physical component summary (F = 3.887; *p* = 0.056; $\eta_{p}^{2}$ = 0.095; 1 − *β* = 0.484) or mental component summary (F = 0.424; *p* = 0.519; $\eta_{p}^{2}$ = 0.011; 1 − *β* = 0.097). For the CRT group, the two-way RM-ANOVA revealed a significant effect of time on physical functioning (F = 25.465; *p* < 0.001; $\eta_{p}^{2}$ = 0.408; 1 − *β* = 0.998), general health (F = 4.189; *p* = 0.048; $\eta_{p}^{2}$ = 0.102; 1 − *β* = 0.513), vitality (F = 15.209; *p* < 0.001; $\eta_{p}^{2}$ = 0.291; 1 − *β* = 0.967), role emotional (F = 4.329; *p* = 0.044; $\eta_{p}^{2}$ = 0.105; 1 − *β* = 0.527) and physical component summary (F = 12.973; *p* = 0.001; $\eta_{p}^{2}$ = 0.270; 1 − *β* = 0.939), but there were no changes in role physical (F = 1.293; *p* = 0.263; $\eta_{p}^{2}$ = 0.034; 1 − *β* = 0.198), bodily pain (F = 2.325; *p* = 0.136; $\eta_{p}^{2}$ = 0.059; 1 − *β* = 0.318), social functioning (F = 1.761; *p* = 0.193; $\eta_{p}^{2}$ = 0.045; 1 − *β* = 0.253), mental health (F = 2.728; *p* = 0.107; $\eta_{p}^{2}$ = 0.069; 1 − *β* = 0.363) or mental component summary (F = 1.542; *p* = 0.222; $\eta_{p}^{2}$ = 0.040; 1 − *β* = 0.227). No changes were observed in the scores of any of the subscales or summary components for the CG. One-way ANOVA showed that the change in CRT group was greater than in the CG (*p* < 0.001; *d* = 2.09) in the physical functioning score.

### 3.2. Timed Up and Go

Time for the TUG test is presented in Table 3. Two-way RM-ANOVA revealed a significant effect of time for the TRT (F = 11.003; *p* = 0.002; $\eta_{p}^{2}$ = 0.229; 1 − *β* = 0.898) and CRT (F = 35.968; *p* < 0.001; $\eta_{p}^{2}$ = 0.493; 1 − *β* = 1.000) groups. There were no changes in the CG (F = 0.005; *p* = 0.942; $\eta_{p}^{2}$ = 0.000; 1 − *β* = 0.051). One-way ANOVA showed that the decrease in the CRT group was greater than in the CG (*p* < 0.001; *d* = 0.34).

## 4. Discussion

Biological aging is associated with structural and functional deterioration in most physiological and psychological systems, which negatively impacts activities of daily living and HRQoL perceptions in older adults [1]. However, this objective and subjective deterioration can be countered or reduced with exercise. In this regard, the present study shows that 12 weeks of heavy-resistance circuit training leads to significant improvements in perception of HRQoL as well as significant improvements in functional mobility in healthy older women. From a practical point of view, these results are of particular importance for older people, since age-related decreases in muscle strength can negatively affect mobility, which, together with loss of balance, increases the risk of falling [21]. Furthermore, our results show that CRT, but not TRT, elicited improvements in HRQoL perceptions, suggesting that a greater potential of CRT promotes not only objective but also subjective health status, which may help older people self-assess adherence to CRT programs.

Health-related quality of life is a subjective concept, indirectly measurable by means of a questionnaire composed of a set of dimensions, each of which contributes to quantifying some relevant feature or aspect of this concept. In the present study, we used the Spanish version of the SF-36 Health Survey questionnaire [15], which comprises eight dimensions that in turn define two main components of health: physical and mental component summary. In the CRT group, statistically significant changes were observed in four of the eight dimensions: physical function, general health, vitality and role emotional, and in physical component summary. In contrast, statistically significant changes were only observed in one dimension (i.e., physical function) in the TRT group, and no statistically significant changes were observed in any of the dimensions comprising the questionnaire in the CG. It is relevant to note that a 12-week intervention produced not only an improvement at the physical level, as previously reported by other authors [13,22,23,24], but also an improvement in a psychological variable, the perception of HRQoL, in the CRT group. This effect may contribute to good adherence to the program by participants.

Several studies have examined the effect of traditional heavy-resistance training on the perception of quality-of-life parameters in older people and published results similar to those of the present study. Cassilhas et al. [25] assessed whether the intensity of a 24-week resistance training program might affect the perception of HRQoL, and they observed that the group that trained at an intensity of 50% of 1RM improved some dimensions related to HRQoL as assessed with the SF-36 questionnaire, while the group that trained at intensities of 80% of 1RM showed no changes. Another study that assessed the effects of heavy-resistance training on the perception of HRQoL was conducted by Sillanpää et al. [14]. In that study, they established four groups: a control group, a cycle ergometer endurance group, a heavy-resistance group and a group that combined both training programs. After 21 weeks of training, statistically significant improvements were observed in several dimensions of the SF-36 questionnaire in the endurance training group and in the group that combined both types of training, with no variations observed in the heavy-resistance group or in the control group. Sillanpää et al. [14] observed that only heavy-resistance training improved the perception of HRQoL when combined with aerobic exercise. These authors observed that there were correlations between cardiorespiratory fitness and some of the dimensions of HRQoL. This is in accordance with the findings of the present study. The arrangement of exercises in circuit training makes the aerobic component of the session very high [11,12,13]. This seems to be determinant in achieving the results obtained on the perception of quality of life assessed with the SF-36 questionnaire.

Regarding functional mobility, the intervention resulted in a statistically significant improvement in the TUG test in both experimental groups, with no changes observed in the CG. These results are in line with previous studies [26,27,28,29,30] that support the functional benefits that older people can obtain from several months of resistance training when training loads are heavy. In 2005, Hess et al. [28] subjected a group of octogenarians to a resistance training program at 80% of 1RM, three times per week; after 10 weeks of training, participants improved their performance on the TUG test by 15.7%. Fiatarone et al. [26] demonstrated that resistance training raised functional mobility even in people over 90 years old. The results of the present study support the theory that heavy-resistance training improves functional mobility. Furthermore, only the CRT group reported differences with the CG; this may be due to the exercise’s configuration in circuit. The CRT group continuously changed exercise, having to move from one exercise to another in a short period of time (i.e., ~35 s), in contrast to the subjects in the traditional group, who waited in place for the rest time to pass until the next set was performed (i.e., 180 s).

Mobility problems are of relevance in the elderly, as they are a risk factor for falls. Consequently, both muscle power and strength are important determinants of mobility, and resistance training is a powerful tool to induce specific neuromuscular adaptations that translate into improved mobility in healthy older adults [29]. Participants in the CRT and TRT groups in the present study reduced the time taken to perform the TUG, which, according to Sai et al. [31], may indicate an improvement in dynamic balance and a reduction in the risk of falling, as the TUG test is both sensitive and specific in identifying individuals with balance disorders who are likely to fall. Shumway-Cook et al. [32] have established cutoff values to cluster subpopulations of older adults according to their mobility status, and they reported that older adults with scores of greater than 13.5 s performing the TUG test have a 90% probability of being fallers. In both experimental groups, a mean pre-training TUG score of 10.38 s corresponded to a fall risk of approximately 20%. After 12 weeks of resistance training, the mean score in the CRT group was 9.24 s and 9.75 s in the TRT group, which corresponds to a risk of falling of ~9% and ~14%, respectively. Therefore, because of resistance training, using the TUG as a measure of fall risk, participants in the experimental groups decreased their fall risk by ~6–11%. Although participants at a high risk of falls were not used in this study, improvements in functional mobility may lead to a later onset of loss of autonomy and dependence in this population [33].

Finally, we acknowledge some limitations of this study: the sample size was limited, resulting in a small effect size of the results. For this reason, our results cannot be generalized to other types of populations. With regard to the methodological procedures used in the present study, the lack of an evaluation of other physiological measures (e.g., muscle strength and power, exercise metabolism measured by gas analysis or body composition) that would provide a more comprehensive picture of the training effect may also be considered as a potential limitation. Despite these limitations, this study provides important insights into how heavy-resistance training is well-tolerated and improves mobility and perception of HRQoL in older women.

## 5. Conclusions

Heavy-resistance training can offer physicians a viable option to improve functional performance, helping their patients stay independent, active and healthy. The present results demonstrate that CRT can be as effective as TRT for improving objective health factors such as functional mobility, but is more effective at increasing subjective health, as it improves perception of HRQoL in untrained older women. The present results suggest that CRT may be a time- (and hence cost-) effective way to elicit multiple positive functional and psychological adaptations in this population.

## Figures and Tables

**Table 1:** Pre-training characteristics of the participants in each training group. Values are given as mean ± SD.

	TRT (*n* = 15)	CRT (*n* = 15)	CG (*n* = 10)
Age (year)	64.2 ± 4.0	64.7 ± 4.4	63.7 ± 2.1
Weight (kg)	71.2 ± 8.0	66.8 ± 6.6	71.6 ± 9.9
Height (cm)	153.0 ± 5.2	152.9 ± 6.8	155.5 ± 4.8
BMI (kg·m^−2^)	30.5 ± 4.1	28.7 ± 3.6	29.7 ± 5.1

TRT: traditional resistance training group; CRT: circuit resistance training group; CG: control group; BMI: body mass index.

**Table 2:** Pre- and post-training values of perception of HRQoL. Values are given as mean ± SD.

	TRT			CRT			CG		
	Pre	Post	%Δ	Pre	Post	%Δ	Pre	Post	%Δ
Physical functioning	78.0 ± 17.5	86.0 ± 10.4 †	6.7	79.3 ± 16.8	91.0 ± 10.9 †	13.0 ‡	78.5 ± 14.3	76.5 ± 19.4	−2.0
Role physical	96.0 ± 13.0	100.0 ± 0.0	4.0	95.7 ± 9.0	98.3 ± 6.5	2.7	80.0 ± 32.9	80.0 ± 32.9	0.0
Bodily pain	69.3 ± 22.6	71.7 ± 18.8	2.3	67.6 ± 23.2	74.7 ± 19.5	7.1	65.7 ± 25.5	72.4 ± 22.9	6.7
General health	66.1 ± 17.1	73.0 ± 14.7	6.9	63.1 ± 18.3	73.1 ± 19.6 †	8.1	60.0 ± 114.7	66.5 ± 13.6	6.5
Vitality	65.7 ± 19.4	73.0 ± 16.4	7.3	65.3 ± 23.5	83.0 ± 16.5 †	17.7	68.0 ± 22.9	69.5 ± 18.5	1.5
Social functioning	85.1 ± 21.7	93.5 ± 11.3	8.3	86.0 ± 20.3	91.7 ± 17.5	5.7	88.0 ± 23.4	92.6 ± 19.6	3.8
Role emotional	97.8 ± 8.5	97.8 ± 8.5	0.0	91.1 ± 15.3	97.8 ± 8.5 †	6.7	88.3 ± 19.4	91.6 ± 18.1	3.3
Mental health	74.9 ± 21.8	80.8 ± 15.4	5.9	72.5 ± 18.2	80.5 ± 20.6	8.0	72.0 ± 18.8	78.8 ± 16.5	6.8
Physical component summary	47.7 ± 7.2	51.1 ± 5.2	3.5	46.7 ± 9.4	53.1 ± 6.3 †	6.3	48.4 ± 9.4	48.3 ± 9.9	−0.1
Mental component summary	49.9 ± 11.9	52.0 ± 9.9	2.1	49.5 ± 9.6	53.6 ± 7.7	4.1	46.5 ± 12.2	51.4 ± 12.3	4.9

TRT: traditional resistance training group; CRT: circuit resistance training group; CG: control group; ∆: change; †: statistically significant difference from pre- to post-training (*p* < 0.05); ‡: statistically significant difference from CG (*p* < 0.05).

**Table 3:** Timed Up and Go data. Values are given as mean ± SD.

	TRT			CRT			CG		
	Pre	Post	%Δ	Pre	Post	%Δ	Pre	Post	%Δ
Time (s)	10.38 ± 1.67	9.75 ± 1.33 †	−5.4	10.38 ± 1.15	9.24 ± 0.95 †	−10.7 ‡	10.32 ± 1.11	10.34 ± 1.02	0.28

TRT: traditional resistance training group; CRT: circuit resistance training group; CG: control group; ∆: change; †: statistically significant difference from pre- to post-training (*p* < 0.05); ‡: statistically significant difference from CG (*p* < 0.05).

## Data Availability

The datasets used during the current study are available from the corresponding authors on reasonable request.

## Supplementary Materials

The following supporting information can be downloaded at: https://www.mdpi.com/article/10.3390/biology11050626/s1. Figure S1. Evolution of the loads for each exercise over the period of training.
