# Supplementary figures and images for: Does Heavy-Resistance Training Improve Mobility and Perception of Quality of Life in Older Women?
Source: Biology (Basel). 2022 Apr 20;11(5):626. doi: 10.3390/biology11050626 (PMC9137969; doi:10.3390/biology11050626)

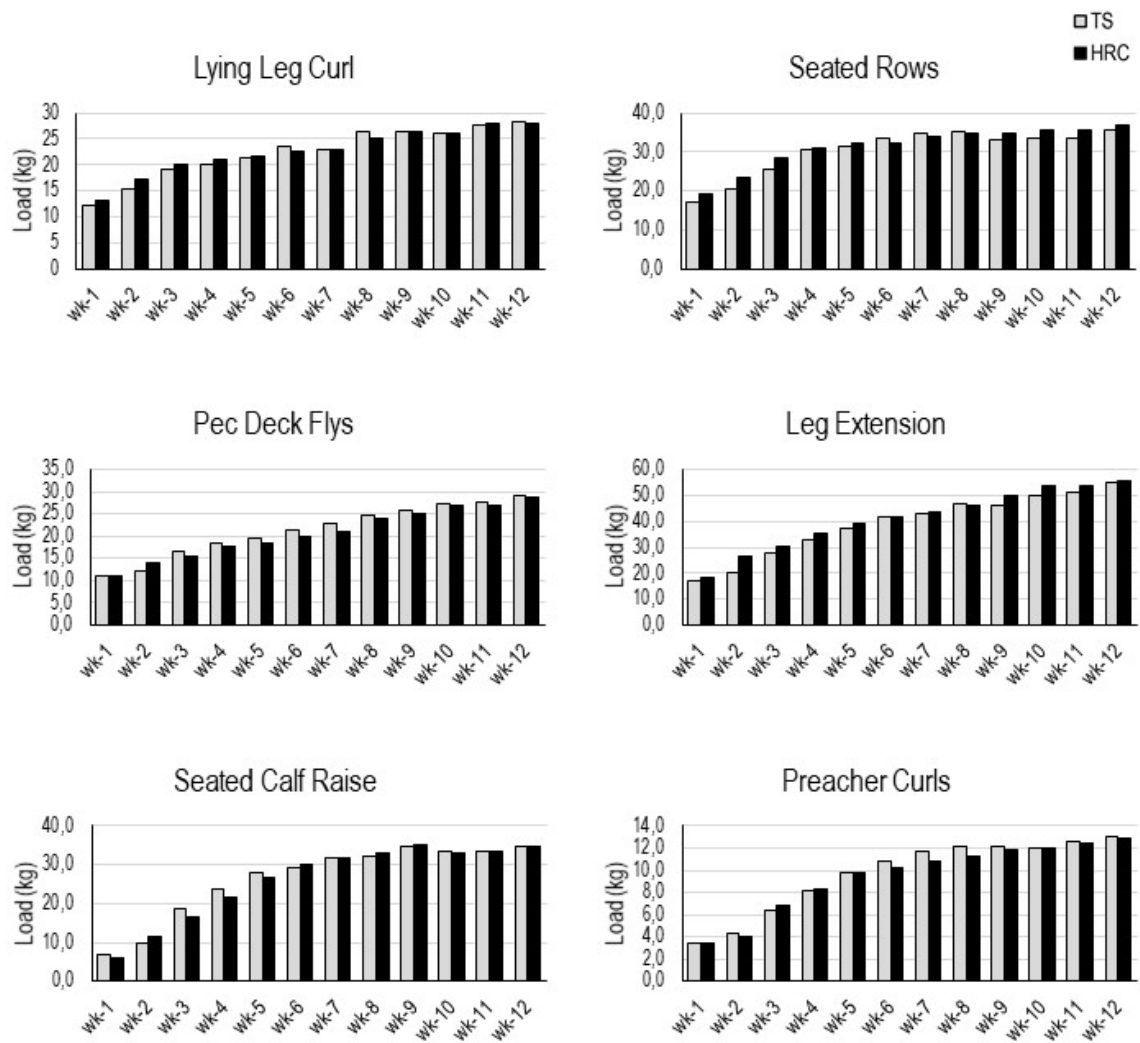

Figure S1. Evolution of the loads for each exercise over the period of training.

Supplement: Supplementary file 1 [file biology-11-00626-s001.zip › biology-1669720-supplementary.pdf]
